# The High Permeability of Nanocarriers Crossing the Enterocyte Layer by Regulation of the Surface Zonal Pattern

**DOI:** 10.3390/molecules25040919

**Published:** 2020-02-19

**Authors:** Ya-Nan Chang, Yuelan Liang, Shibo Xia, Xue Bai, Jiaxin Zhang, Jianglong Kong, Kui Chen, Juan Li, Gengmei Xing

**Affiliations:** 1 CAS Key Laboratory for Biomedical Effects of Nanomaterial and Nanosafety, Institute of High Energy Physics, Chinese Academy of Sciences, Beijing 100049, China; changyn@ihep.ac.cn (Y.-N.C.); liangyl@ihep.ac.cn (Y.L.); xiasb@ihep.ac.cn (S.X.); xbai@ihep.ac.cn (X.B.); zhangjiaxin@ihep.ac.cn (J.Z.); kongjl@ihep.ac.cn (J.K.); chenkui@ihep.ac.cn (K.C.); lijuan@ihep.ac.cn (J.L.); 2 Institute of Physical Science and Information Technology, Anhui University, Hefei 230601, China

**Keywords:** gold nanoparticles, surface pattern, oral administration, intestinal epithelium, permeability

## Abstract

The intestinal epithelium is a major barrier that limits the absorption of oral drugs. The integrity of the epithelial tissue is a very important factor for preventing intestinal diseases. However, destabilization of the epithelium can promote the transportation of nanocarriers and increase the absorption of oral drugs. In our research, three different gold nanoparticles (GNPs) of the same size but with differing negative surface charge were designed and constructed as a model to determine the surface properties crucial for promoting absorptivity and bioavailability of the nanocarriers. The higher the ratio of surface carboxyl groups on GNPs, the higher capacity to induce transepithelial electrical resistance change and cell monolayer tight junction opening with higher permeability. The half carboxyl and half methyl surfaced GNPs displayed unique zonal surface patterns exhibited the greater ability to pass through intestinal epithelial cell layer but had a relatively small influence on tight junction distribution.

## 1. Introduction

Oral administration of drugs is often preferred over the parenteral route due to its convenience, safety, and reduced health care costs [1]. An intact intestinal epithelium, unstirred water layer, tight junctional complex between cells, and polarized cell membrane have the natural capacity to prevent permeation of exogenous substances (e.g., bacteria, toxins, food antigens, and carcinogens) and protect the human body [2]. Epithelial cell tight junctions are an important component of the intestinal mucosal barrier. Once tight junctions are impaired, permeability increases between intestinal cells, thereby allowing bacteria, endotoxins, and macromolecular substances to enter the circulation system [3]. Many intestinal diseases are associated with the destruction of the intestinal epithelium, including inflammatory bowel disease [4,5], infectious diarrhea [6], and intestinal tumors [7]. Therefore, classic issues in the research of oral nanocarrier preparation are still how to enhance absorptivity and ensure biosafety. Nanostructure drug delivery systems have been designed to promote drug transport through the intestinal barrier [8]. Many factors, including size [9], surface charge [10,11], hydrophobicity [12], and concentration of nanoparticles, can influence permeation through the epithelial cell monolayer [13]. The intestinal epithelium and its mucosal layer can limit permeation of drugs with high molecular weight or polarity [14]. Generally, an ideal drug delivery system should not only enhance absorption of the drug but also ensure intestinal epithelium safety.

Drug molecules cross through the enterocyte monolayer by transcellular pathways and paracellular flux [1,15]. The space between adjacent endothelial cells increases to at least 1.2–2 µm, resulting in permeability for drug delivery [16]. Nanodelivery technology was designed to cross through the intestinal epithelial layer and explicitly but transiently disrupt intercellular junctions [17]. Surface properties are critical for the safety and biological effects of a nanocarrier. In the current research, we designed a series of gold nanoparticles (GNPs) with different negative surface charges, which were variable dispersity under different pH conditions. We further investigated whether these GNPs could effectively cross the intestinal epithelial layer and clarified the potential mechanism by which absorption was increased but intestinal epithelial integrity was maintained [18]. Therefore, we established a Caco-2 cell monolayer, used previously to study gut absorption of nanostructures in vitro [17,19], to model the intestinal epithelial cell layer. This study should provide insight into achieving the balance between optimal intestinal safety and higher permeability of nanoparticles utilized for the delivery of orally administered drugs.

## 2. Results and Discussions

### 2.1. GNP Synthesis and Modification

**Figure 1:**
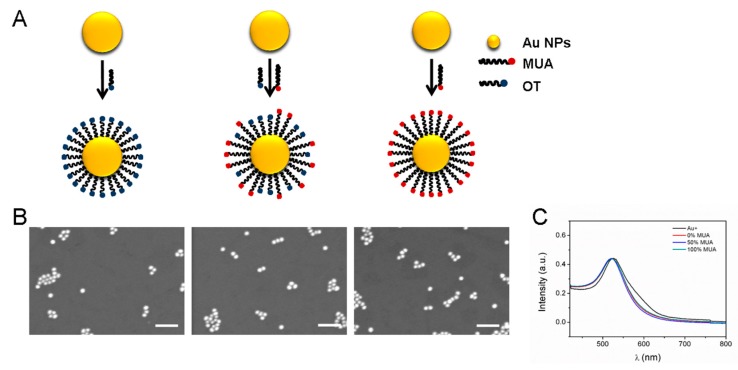
Characterization of gold nanoparticles (GNPs) with different surface modifications. (**A**) Schematic of GNP surface modifications with 0% MUA, 50% MUA, and 100% MUA. (**B**) SEM images of different surface-modified GNPs. Scale bar is 100 µm. (**C**) Ultraviolet-visible spectra of GNPs before and after surface modification.

To understand the impact of surface properties on interactions between GNPs and the intestinal epithelium, we synthesized 15-nm GNPs grafted with a self-assembled monolayer containing 1-octanethiol (OT) and/or 11-mercaptoundecanoic acid (MUA) using established techniques [20,21] (Figure 1A). Hydrophilic MUA- and OT-modified GNP surfaces have been reported to show the least harmful effects in vivo and in vitro [22]. The GNPs with surfaces modified with different ratios of MUA:OT (1:0, 1:1, and 0:1) [22] were named 0%, 50%, and 100% MUA GNPs, respectively. Scanning electron microscopy images confirmed that the GNPs were spherical with a diameter of about 15 nm (Figure 1B and Table 1). Ultraviolet (UV)-visible spectra showed that the three synthesized nanoparticles had a uniform size and good dispersion (Figure 1C). The zeta potentials of the GNPs were −4.50 ± 2.25 mV, −17.52 ± 0.25 mV, and −27.50 ± 0.25 mV, respectively. Accompanied by the increasing proportion of MUA on the surface, the zeta potential absolute values gradually increased (∣Z_0%MUA-GNPs_∣ < ∣Z_50%MUA-GNPs_∣ < ∣Z_100%MUA-GNPs_∣) (Table 1). The toxicity of GNPs is dependent on many factors, including size and concentration [9]. Here, we measured the cell survival rate after cells were exposed to the three GNPs at different concentrations for 24 h (Figure S1). Based on our results, we chose a concentration of 0.05 mg/mL as it did not influence the viability of the treated Caco-2 cells.

### 2.2. Stability of GNPs in Gastrointestinal Environments

**Figure 2:**
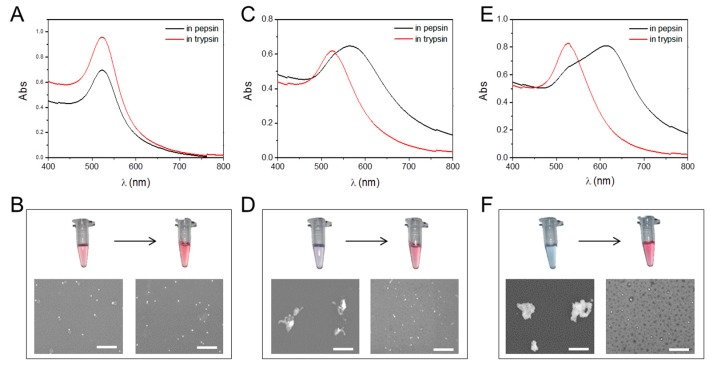
Aggregation of GNPs with different surface modifications under different pH values and enzymes present in solution. (**A**) Ultraviolet-visible spectrum of 0% MUA GNPs in pepsin and trypsin. (**B**) Photo and SEM images of 0% MUA GNPs in pepsin and trypsin. (**C**) Ultraviolet-visible spectrum of 50% MUA GNPs in pepsin and trypsin. (**D**) Photo and SEM images of 50% MUA GNPs in pepsin and trypsin. (**E**) Ultraviolet-visible spectrum of 100% MUA GNPs in pepsin and trypsin. (**F**) Photo and SEM images of 100% MUA GNPs in pepsin and trypsin. Scale bar is 500 µm.

As an oral drug carrier, GNPs need to pass through various biological environments within the gastrointestinal tract. Thus, we first examined if the properties of the GNPs changed under different gastrointestinal environments. Results showed that the GNPs aggregated in the simulated gastric environment (HCl, with pepsin, pH = 1.2), and the degree of aggregation increased as the number of carboxyl groups increased (Figure 2). The UV spectrum images showed the extent of particle aggregation. The UV spectral peak of the 0% MUA GNPs in simulated gastric fluid (with pepsin, pH = 1.2) was observed at 520 nm and did not vary in simulated intestinal fluid (with trypsin, pH = 7.0) (Figure 2A). The red-wine solution color was an indication of well-dispersed particles, as was also observed in the SEM images (Figure 2B). The UV spectral peak of the 50% MUA GNPs was at 569 nm in the simulated gastric fluid and at 520 nm in the simulated intestinal fluid, showing that the GNPs were aggregated in the gastric fluid but re-dispersed in the intestinal fluid (Figure 2C). The solution color of the 50% MUA GNPs was purple red in simulated gastric fluid but wine red when the aggregated GNPs were added into the simulated intestinal fluid. The color change showed the process of aggregation and re-dispersion, with the same result seen in the SEM images (Figure 2D). Although the 100% MUA GNPs exhibited similar properties as the 50% MUA GNPs, aggregation was greater, as shown by the UV spectral peak detected at 614 nm (Figure 2E) and blue color of the solution in the simulated gastric fluid, suggesting the aggregation of particles. The SEM images also showed the same result (Figure 2F). This deformation property of the GNPs, i.e., aggregation in gastric fluid and re-dispersion in intestinal fluid, should protect the nanodrug carrier from gastric fluid digestion [23,24] but ensure monodispersal when in the intestinal tract.

### 2.3. Constructing Model of Caco-2 Cell Monolayer

**Figure 3:**
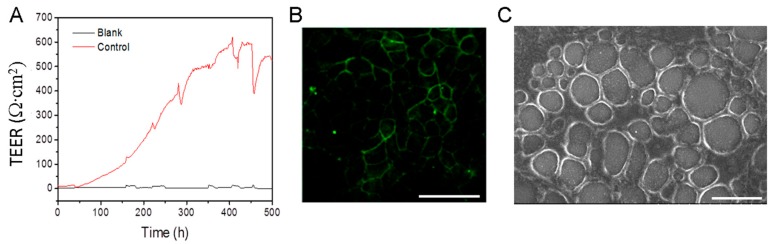
Formation of Caco-2 cell monolayer. (**A**) Continuous transepithelial electrical resistance (TEER) analysis of Caco-2 cell monolayer over 21 d. (**B**) Caco-2 cell monolayer formed after 21 d of culture. Green is immunofluorescence stained ZO-1 protein (tight junction protein). Scale bar is 40 µm. (**C**) SEM image of Caco-2 cell monolayer. Scale bar is 20 µm.

To replace animal models in drug research due to animal welfare and cost and time considerations, human intestinal function models were developed. These in vitro static and dynamic models are constructed by inserting a polycarbonate membrane in a microfluidic device to support the culture of Caco-2 cells to form an epithelial monolayer (transepithelial barrier) [17,19]. To validate the permeability of the GNPs passing through the cell monolayer, we utilized the Transwell static model and microfluidic chip dynamic model, respectively, in our experiment. In these models, the Caco-2 cells were cultured for at least 21 d so that the transepithelial electrical resistance (TEER) increased to 500 Ω·× cm^2^ (Figure 3A), and the cells differentiated to form monolayers and spontaneously exhibited enterocyte-like phenotypes, such as brush borders and tight intercellular junctions on the surface [19]. The tight junction protein ZO-1 in the Caco-2 cell monolayers was stained with green fluorescence antibodies (Figure 3B) to ensure that the regular-shaped intestinal epithelial cell monolayer with tight junctions were visible under laser confocal microscopy. The SEM images showed the cell monolayer details (Figure 3C) and that the Caco-2 cells had formed regular tight junctions.

### 2.4. Permeability of GNPs in Flowing Fluid

**Figure 4:**
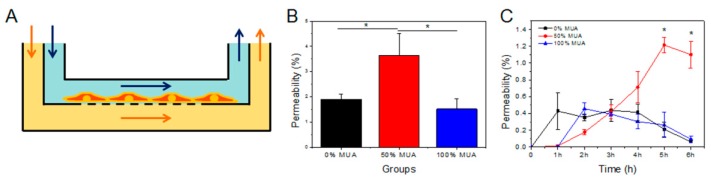
Permeability of GNPs with different surface modifications in the microfluidic chip. (**A**) Human gut-on-a-chip. Schematic of the gut-on-a-chip device showing a flexible porous polylysine-coated membrane lined by gut epithelial cells crossed horizontally through the microchannel. (**B**) Aggregate permeability of the three GNPs tested by the microfluidic chip over 6 h. * *p* < 0.05. (**C**) Permeability of the three GNPs tested by the microfluidic chip each hour over 6 h.

The Caco-2 cell monolayer model with microfluidic device is portrayed in Figure 4A. To mimic the physiological microenvironment of intestinal absorption, fluid flowed slowly and cyclically into the top and bottom channels of the device, which were segmented by a polycarbonate membrane (1-µm pores) upon which the Caco-2 cell monolayer grew [18]. In the device, the Caco-2 cell monolayer was treated by a continuously flowing fluid containing the GNPs at the same concentration. The GNPs in the bottom channels were collected at 1 h intervals for 6 h, and the GNPs that crossed the cell monolayer were quantified using inductively coupled plasma mass spectrometry (ICP-MS). Permeability was determined as the rate of GNPs collected from the bottom channel compared to the dosing amount in the upper channel. After 6 h, total permeability of the 50% MUA GNPs (4.15 ± 0.87%) was the highest among the three GNPs. Compared with the 0% and 100% MUA GNPs, the 50% MUA GNPs exhibited the highest permeability and the difference was significant (*p < 0.05*) (Figure 4B). The permeability of the 50% MUA GNPs peaked at 5 h, with the increase from 0 to 5 h showing a 0.29 slope (y = 0.2937 × −0.3739, R^2^ = 0.9556). The permeability peaks of the 0% and 100% MUA GNPs were at 3 h and 2 h, respectively, and their peak values were only about one third that of the 50% MUA GNPs (Figure 4C). The GNPs were uniform in size but their OT- and MUA-modified surface physicochemical properties were distinct. The permeability of the 50% MUA GNPs in the microfluidic chip demonstrated a sustained and linear growth curve from 0 to 5 h, indicating that the 50% MUA GNPs exhibited unique properties allowing permeation of the Caco-2 cell monolayer. We further evaluated their intestinal safety and investigated why and how these particles achieved permeability, respectively.

### 2.5. Permeability of GNPs in Static State

**Figure 5:**
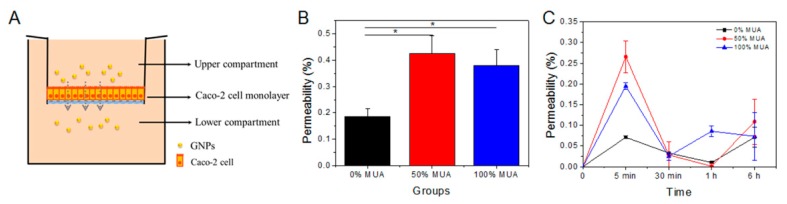
Permeability of GNPs with different surface charge distributions at various time points. (**A**) Schematic of GNP transportation in the transwell chamber from the upper to lower compartment. (**B**) Permeability of the three GNPs after 6 h. * *p* < 0.05. (**C**) Permeability of the three GNPs at 5 min, 30 min, 1 h, and 6 h.

Using the static state Transwell model, we compared the permeability of the different GNPs. Transmittance was calculated by comparing the final number of GNPs in the lower chamber to the total amount added in the upper chamber (Figure 5A). Total permeability rates were 0.19 ± 0.03%, 0.42 ± 0.07%, and 0.38 ± 0.06% following treatment (6 h) with the 0%, 50%, and 100% MUA GNPs, respectively (Figure 5B). Comparison showed that the 50% and 100% MUA GNPs were significantly different from the 0% MUA GNPs, whereas there was no evident difference between them (Figure 5B). The 50% MUA GNPs induced the highest total permeability, whereas the 0% MUA GNPs showed the lowest capacity for crossing the cell monolayer. As shown in Figure 5C, all GNPs reached peak permeability within 5 min and then declined, though the 100% MUA GNPs presented a second peak at 1 h after treatment. The lowest permeability point appeared at 1 h in the 0% and 50% MUA GNP treatments, whereas the lowest point for the 100% MUA GNPs was at 30 min, though the absolute value of the lowest point was higher than that for the other two particles. Although the permeability trends of the GNPs were distinct, the 50% and 100% MUA GNPs exhibited greater fluctuation in permeability than the 0% MUA GNPs.

### 2.6. Distinct Capacity of GNPs in Modulating Caco-2 Cell Monolayer TEER

**Figure 6:**
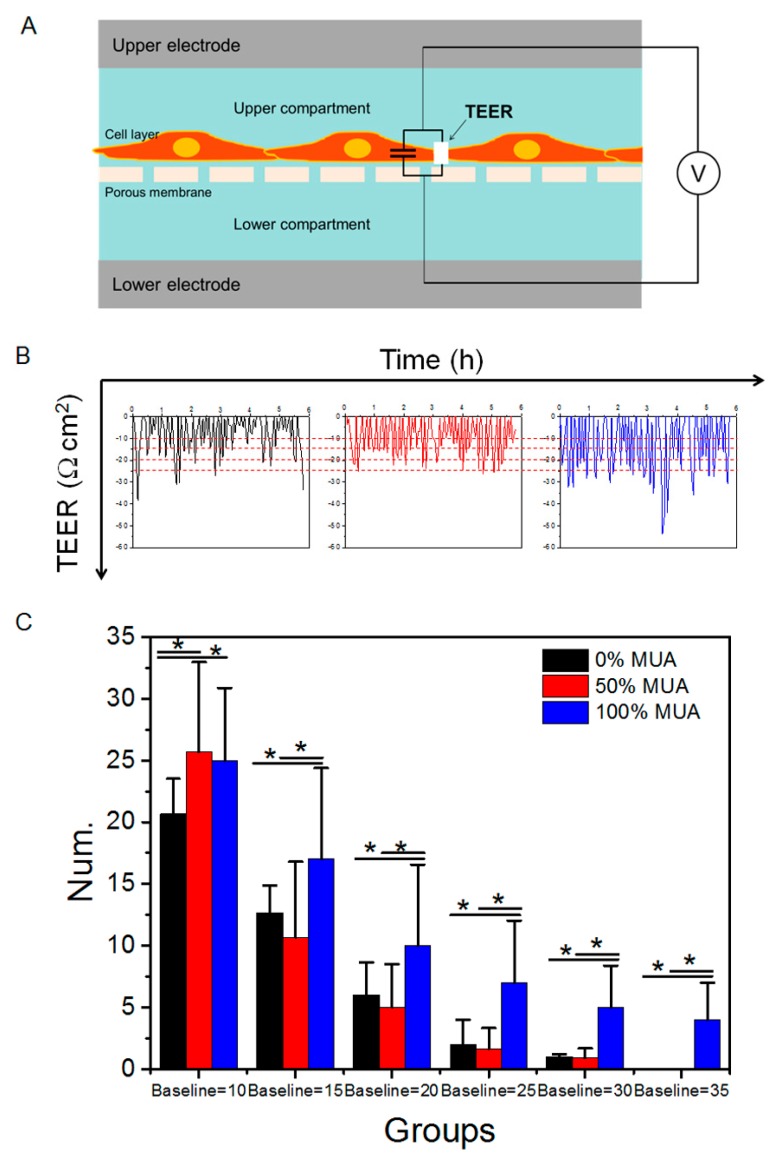
TEER analysis of Caco-2 cell monolayer treated with different surface-modified GNPs. (**A**) Schematic of TEER test mechanism. (**B**) TEER test curve after exposure to GNPs for 6 h. Red line is chosen baseline. (**C**) Statistical analysis of TEER curve after 6 h. * *p* < 0.05.

TEER is a very sensitive and reliable measure of electrical resistance across a cellular monolayer and can be used to confirm monolayer integrity and permeability [25]. Changes in TEER are also widely used to dynamically and quantificationally describe tight junction opening in cell monolayers (Figure 6A). We measured the real-time TEER of the cell monolayers treated by the three GNPs and then analyzed the varying TEER curves after 6 h of treatment (Figure 6B). TEER changes mainly reflect ionic conductance of the paracellular pathway in the epithelial monolayer, whereas the flux of non-electrolyte tracers (expressed as the permeability coefficient) indicates the paracellular water flow, as well as tight junction integrity [26]. Therefore, the declining TEER curves in the current study were an indication of tight junction disruption and that GNPs crossed the cell barrier by the paracellular pathway. To eliminate false positive changes caused by background noise, every pulse (declined value greater than 10 Ω × cm^2^) in the TEER curves was recorded and calculated (Figure 6B). After more than 20 experimental repeats, statistical analysis showed that the number of troughs in the TEER curve of the cell monolayer increased obviously for the 50% and 100% MUA GNPs following treatment (6 h), whereas the 0% MUA GNPs exhibited the lowest capacity to induce TEER decline. By gradually increasing the numerical baseline of the TEER curve amplitude (15, 20, 25, 30, and 35 Ω × cm^2^, respectively), there were significantly more TEER curve troughs for the 100% MUA GNPs than for the 0% and 50% MUA GNPs, and this trend persisted from 15 to 35 Ω × cm^2^ (Figure 6C). These results indicated that the 100% MUA GNPs demonstrated the strongest capacity to induce tight junction opening and, thus, facilitated the paracellular permeation of the GNPs. Increasing transport by the paracellular pathway is an advantage of nanoscale drug delivery to increase the permeability of particles [27]; however, tight junction opening likely disturbs the stability of Caco-2 cell monolayers [28].

### 2.7. GNPs Modulate Structural Changes of Tight Junctions in Caco-2 Cell Monolayers

**Figure 7:**
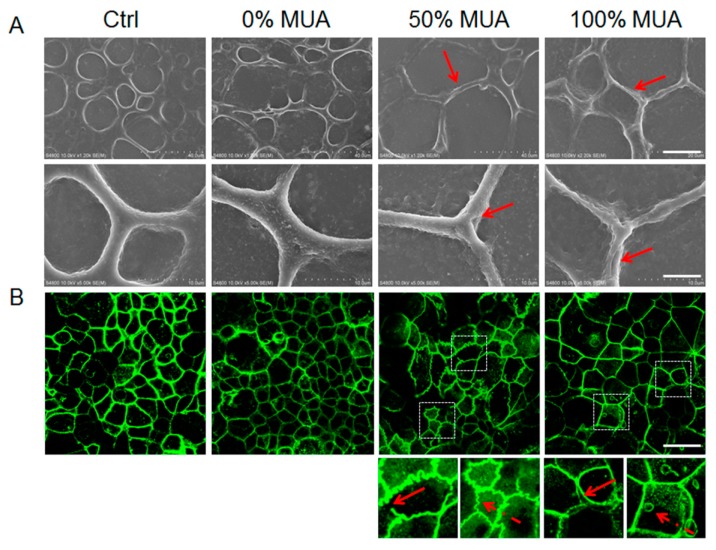
Disrupted tight junctions impacted by different surface-modified GNPs. (**A**) SEM images of cell monolayer surface structure. Upper line shows high coverage imaging, scale bar is 20 µm. Lower line shows detailed imaging, scale bar is 5 µm. Red arrows show tight junction details. (**B**) Immunofluorescence imaging of the ZO-1 protein in the Caco-2 cell monolayer and fluorescence intensity of ZO-1 distribution in the Caco-2 cells. Scale bar is 20 µm. Images with white dotted boxes are magnified, as shown under the images, and the red and red dotted arrows show the ZO-1 imaging details.

Tight junctions regulate the paracellular passive diffusion of certain ions and small hydrophilic molecules along concentration gradients crossing through the barrier of cell monolayers [29]. Evaluation of the structural changes of tight junctions is essential for elucidating the mechanism of GNP-induced tight junction opening. Researchers have revealed three distinct sub-components that form tight junction belts, including transmembrane proteins, cytoskeletal elements, and cytoplasmic adaptor proteins that attach the two together [29,30]. We used SEM to observe the tight junction ultrastructure. In the control group, all cells were tightly apposed, and all junctional walls were localized at the intercellular connections forming paracellular belts. As indicated by the red arrows in Figure 7A, the GNPs induced the tight junction protein walls to gradually narrow when the MUA on the GNP surface increased from 0% to 100%. In the 50% MUA GNPs, defective and thin tight junction walls (red arrow) were induced. In contrast, attenuated and obviously furcal tight junction walls (red arrow) were found in the cell monolayer exposed to 100% MUA GNPs, whereas no change in tight junction morphology was found in the 0% MUA GNPs (Figure 7A). These findings support the fact that particles with a more negative surface charge resulted in greater tight junction opening. Zonula occludens proteins (ZO-1, ZO-2, and ZO-3) are typical adaptors in the cytoplasm and can interact directly with tight junction transmembrane proteins and actin in the cytoskeleton [30]. Immunofluorescence imaging of ZO-1 was used to visualize the morphology of the tight junctions in the cell monolayers (Figure 7B). In the control group, continuous rings of ZO-1 labeled by green fluorescent antibodies were observed around the monolayer cells. Compared with the control, the fluorescence signal of ZO-1 was obviously weakened by the 0% MUA GNPs, but ultrastructural alterations of tight junctions were not observed. As shown in Figure 7B, discontinuous (red arrow in lower left corner of magnified image) and corrugated (red dotted arrow in lower right corner of magnified image) tight junctions were found between cells in the 50% MUA GNP group. More critically, the 100% MUA GNPs induced intercellular spaces (red arrow in lower left corner of magnified image) and ZO-1 labeling vesicles in the cytoplasm (red dotted arrow in lower right corner of magnified image) (Figure 7B). These results suggest that the 100% MUA GNPs markedly altered the distribution of the ZO-1 protein within the cells.

To determine whether the varied morphology of the tight junctions was due to changes in tight junction protein expression, proteins in the treated cells, including claudins, ZO-1, and villin, were analysed by Western blotting (Figure S2). Densitometric analysis demonstrated that various surface modifications endowed the GNPs with distinct activity, and all GNPs caused a decrease in protein expression, though there were no significant differences compared with the control group. This suggests that the TEER fluctuations of the cell monolayer treated by GNPs might not be related to the expression of these proteins. In enterocyte systems, tight junctions form a semipermeable paracellular diffusion barrier, which allows ion- and size-selective passive diffusion. All GNPs in our experiment were the same size (Figure 1C) but had varied surface charges (Table 1). The OT chain has a hydrophobic methyl end, which can freely enter a membrane’s hydrophobic interior [31]. Here, the 0% MUA GNPs, with surfaces modified by OT, were transported through the cell monolayer via transcellular pathways. The small TEER amplitude and negligible alteration in the ultrastructure of the tight junctions further confirmed that the junctional complexes restricted the passive diffusion of particles through the paracellular pathway [30] (Figure 6B and Figure 7A,B).

Due to their negative surface charge, carboxyl nanoparticles can deprive Ca^2+^ from cadherins at adherens junctions and thus disrupt tight junction assembly [1,32]. In particular, the ultrastructure of tight junctions, furcal tight junctions (Figure 7A), and intercellular spaces (Figure 7B) imply that the 100% MUA GNPs, with the most abundant negative surface charge (Table 1), could bind Ca^2+^ and alter tight junction structures, and thus open adherens junctions. Reyes et al. [33] demonstrated that drug-treated cell monolayers induced intercellular spaces and modified junctional tension and suggested that these effects may damage intestinal epithelial integrity, leading to toxicity and disease. The 50% MUA GNPs induced a ruffled ZO-1 belt morphology in the cell monolayer, which has also been reported by Kam et al. [34] and indicates that nanotopography can remodel tight junction proteins and facilitate the paracellular pathway for transport. Here, the surface carboxylated nanoparticles were transported into the target cells mainly through endocytosis [35], whereas ZO-1 was the main component protein of the tight junctions in cytoplasm. Our results implied that the 50% MUA GNPs affected the ZO-1 protein by directly binding to tight junction proteins and actin in the cytoskeleton and facilitating the paracellular pathway. Nevertheless, it was not clear whether this alteration in tight junction structure was derived by direct or indirect interaction between the GNPs and ZO-1. The 50% and 100% MUA GNPs demonstrated the most obvious effects on tight junctions. The highest TEER amplitude indicated that the 100% MUA GNPs strongly regulated the tight junctions and increased paracellular diffusion (Figure 6), but the permeability of the particles was still lower than that of the 50% MUA GNPs. Whether in flowing fluid or in a static state (Figure 4B and 5B), the 50% MUA GNPs demonstrated the highest permeability of all particles, although the particles induced smaller TEER amplitude than that of the 100% MUA GNPs. Thus, the 50% MUA GNPs were likely transported by both transcellular and paracellular pathways through the cell monolayer. The differences in transport mechanism may be related to the surface properties of these particles, with the surface of the 50% MUA GNPs modified by two kinds of molecules (50% MUA and 50% OT) and the surfaces of the other two particles modified by only one kind of molecule (OT or MUA). The hybrid modification using two kinds of molecules with varying lengths (1.2 and 0.9 nm) led to a varied geometrical structure on the particle surface.

In addition, the interaction between the GNPs and cell monolayers can be increased by various reactions, including electrostatic adherence and hydrogen-bond and Van der Waals interactions [36]. Thus, we used atomic force microscopy (AFM) to investigate the geometrical structure of the surfaces of the 50% MUA GNPs.

### 2.8. Surface Characterization of 50% MUA GNPs by AFM

**Figure 8:**
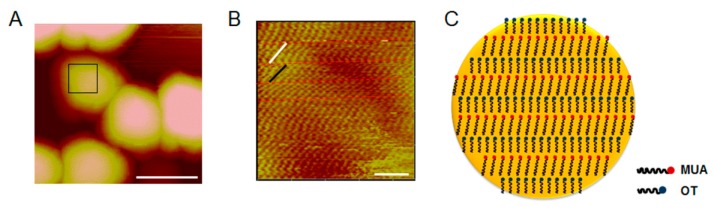
Surface characterization of 50% MUA GNPs by atomic force microscopy (AFM). (**A**) AFM image of 50% 11-mercaptoundecanoic acid (MUA) GNPs. Diameter is ~15 nm. Size of image is 63.88 nm × 63.88 nm. (**B**) AFM image of surface of 50% MUA GNPs. Molecules on surface show regular arrangement with zonal distribution. Size of image is 9.235 nm × 9.235 nm. (**C**) Schematic of the 50% MUA GNP surface pattern.

The magnified AFM images of the surface structure of the 50% MUA GNPs (Figure 8A) indicated that the GNPs exhibited a modified layer of low contrast. Furthermore, the AFM micrographs showed that the GNP surfaces were modified by two different molecules (of different length), which showed regular zonal pattern arrangement on the gold substrate (Figure 8B,C). In the magnified image, different length molecules could be easily distinguished (labeled with red dotted lines). The longer MUA molecule chain is labeled with a white line, whereas the shorter OT molecule chain is labeled with a black line. The lines of each zone were measured in the software, with lengths of 1.2 nm and 0.9 nm (Figure 8B), respectively. The 0% MUA or 100% MUA GNPs were modified with OT or MUA, and their surface structures were uniform.

The zonal surface patterns of the 50% MUA GNPs were constructed using a staggered arrangement of two molecules with different lengths and charge characteristics and regular variation of geometric and charge patterns (Figure 8). Due to the rough surface and rhythmic variety of the hydrophilic and hydrophobic molecules on the surface, the 50% MUA GNPs in fluid flow possessed more opportunities to attach to the cell monolayer surface and induce various interactions, and then cross the Caco-2 cell monolayer by different pathways.

## 3. Material and Methods

### 3.1. Gold Nanoparticles Synthesis and Modification

Synthesis of GNPs was carried out using citrate reduction as previously reported [37]. To investigate the effects of GNPs on permeability, three different surface charged types of GNPs by self-assembled monolayer containing 11-mercaptoundecanoic acid (MUA) and 1-octanethiol (OT), including 0% MUA GNPs (with 100% OT), 50% MUA GNPs (with 50% MUA and 50% OT), 100% MUA (with 100% MUA) GNPs were used. The GNPs were obtained by exchange of citrate molecules with thiols. Excess thiols were removed by centrifugation for 20 min at 14,400× *g* followed by decantation of supernatants and resuspension in ddH_2_O.

### 3.2. Nanoparticle Characterization

Absorbance spectrum of aqueous suspensions of GNPs was recorded from 400 to 800 nm on a UV-Vis spectrometer (Persee General, Beijing, China). Zeta potential distributions were evaluated using NicompTM 380 DLS particle size analyzer.

### 3.3. GNPs-Enzyme Interaction Measurement

Enzymes (pepsin and trypsin) were dissolved in corresponding solution (HCl and NaCl with pH = 1.2 for pepsin and KH_2_PO_4_ and NaOH with pH = 7.0 for trypsin). GNPs were added into the pepsin solution to 0.5 mg/mL (the same with the concentration in cell assays). After centrifuged at 7000× *g* and the supernatant was discarded, the GNPs were added into trypsin solution. The aggregations of GNPs were measured and determined by UV-Vis spectrometer.

### 3.4. Establishment of an In Vitro Model System for Human Enterocyte and Exposure to GNPs

Caco-2 cells (Human colon adenocarcinoma cell line) were obtained from Cell Culture Center, Institute of Basic Medical Sciences of Chinese Academy of Medical Sciences. After reaching about 80% confluence in 25 cm^2^ flasks, Caco-2 cells were seeded in 24-well Transwell plates at a density of 1 × 10^5^ cells/well. After 21 days growth, the Caco-2 cells monolayer was formed, then GNPs suspensions were added to the culture medium with exposure concentrations at 0.001, 0.01, 0.05, 0.1 µg/mL for various times.

### 3.5. Cell Viability Assay

After 21 days growth, the Caco-2 cells monolayer was formed, then GNPs suspensions were added to the culture medium with exposure concentrations at 0.01, 0.05, 0.1 mg/mL. The Caco-2 cells were treated with different GNPs for various times, then washed with Hank’s Balanced Salt Solution (HBSS) three times and the functional effects to the cells were studied. The cell viability of Caco-2 cells was detected by using a Cell Counting-8 Kit (CCK-8, Dojindo Laboratories). 21 days after cell seeding (1 × 10^5^ cells/insert), Caco-2 cells were treated with GNPs for 12 h. Cells were washed with HBSS three times, and then cultured with CCK-8 reagent for 2 h at 37 °C. The optical density (OD) was measured at 450 nm by a microplate reader (SpectraMax M2).

### 3.6. TEER Measurement

The real-time measurement of integrity of Caco-2 cell monolayer was checked by the transepithelial electrical resistance (TEER) assay using cellZscope (nanoAnalytics GmbH, Münster, Germany). The electrical resistance of Caco-2 intestinal monolayers was measured. For resistance measurements, both apical and basolateral sides of the monolayer were bathed with medium. The cell monolayer with TEER value higher than 500 Ω·× cm^2^ was used in experiment.

### 3.7. Intestinal Monolayer Formation in Chip

To study interaction between nanoparticles and intestinal histodifferentiation in vitro, we cultured human Caco-2 intestinal epithelial cells within a microchannel of a physiological chip microdevice incorporates two layers of parallel microchannels (1 mm wide × 10 mm long × 0.15 mm high) separated by a porous membrane (1 µm pores); the membrane was coated with the polylysine before cell seeding. To establish a confluent monolayer, the cells were plated (1 × 10^5^ cells/cm^2^) on the upper surface of the polylysine-coated porous membrane under constant flow of culture medium (100 µL/h) to mimic the mechanically active microenvironment of living intestine.

### 3.8. Measurement of Monolayer Permeability of GNPs

Caco-2 cells were cultured in 24-well transwell plate for 21 days, followed treated with GNPs at 37 °C for 5 min, 30 min, 1h and 6h. At each time point, we collected the medium in the lower chamber, and then all the mediums were digested with microwave. Gold content in the lysates was measured relative to a serial dilution of gold standard using inductively coupled plasma mass spectrometry (ICP-MS, Thermo Elemental X7, Thermo Science, Waltham, MA, USA). Totle GNPs permeability was normalized by sample treated with buffer.

### 3.9. Tight Junction Protein Expression and Morphologic Observation

Morphologic observation of tight junction was detected by imaging by the laser confocal fluorescence inverted microscope (Nikon, Japan) and scanning electron microscope (SEM, S4800, Japan). After exposed to GNPs, Caco-2 cells were fixed for 20 min at room temperature in 4% paraformaldehyde in PBS and blocked with 5% goat serum and 0.3% Triton X-100 for 60 min. Cells were incubated with mouse anti-ZO-1 (Invitrogen, Carlsbad, CA, USA) at 4 °C overnight, then incubated in FITC-conjugated goat secondary antibody (Abcam, Cambridge, MA, USA) solution for 1 h at room temperature. Cells for SEM imaging were fixed for 20 min at room temperature in 2.5% glutaraldehyde in PBS, then dehydration by gradient ethanol.

### 3.10. Western Blotting

After exposure to the GNPs, the Caco-2 cells were suspended in PBS by scraping and lysed by SDS lysis buffer (Beyotime Biotechnology, Shanghai, China). We used the primary antibody (mouse anti-ZO-1 antibody, 1:200 dilution; mouse anti-villin antibody, 1:200 dilution; rabbit anti-claudin-5 antibody, 1:400 dilution; Abcam) and secondary antibody (HRP labeled goat anti-mouse and goad anti-rabbit IgG, dilution 1:2000, Beyotime Biotechnology). GAPDH was used as a loading control.

### 3.11. Surface Structure of 50% MUA GNPs

GNPs were deposited onto the newly-cleaved mica, and air-dried. The mica was rinsed with deionized water after adsorption for 10 min to remove the free GNPs. The geometrical structure of the surfaces of the GNPs was measured in air by using Atomic Force Microscopy (AFM, Bruker, Billerica, MA, USA), under the AC mode. The scan rate was 4.36 Hz.

### 3.12. Statistical Analysis

All experimental permutations were duplicated and independent experiments were repeated at least in triplicated. The data are presented as the mean ± standard error of three independent experiments. The Student’s t-test was used to calculate the statistical significance. The asterisks * denote *p* values of less than 0.05, and double asterisks ** denote *p* values of less than 0.01 compared to untreated cells, respectively.

## 4. Conclusions

In conclusion, the surface properties of nanoparticles are crucial parameters in regulating biosafety and are closely correlated to interactions between particles and the intestinal cell monolayer. 0% MUA GNPs induced lowest varying TEER, therefore, transported crossing through Caco-2 cell monolayer maybe by transcellular pathways. In total, 100% MUA GNPs triggered high-frequency transient open of TJs of enterocyte layer and accompanied stronger paracellular permeability. On 50% of the MUA GNPs surface, a staggered arrangement of two kinds of molecules (OT and MUA) with varied lengths formed zonal pattern. The zonal pattern and rhythmic variety of hydrophilia and hydrophobic on surface induced 50% MUA GNPs to obtain more opportunities than other GNPs to attach on cell monolayer in static state or flow fluid, therefore, the highest permeability was achieved. The varied geometric construction and negative charge on surface adjust effectively of GNPs crossing through the Caco-2 cells layer may provide a better strategy for designing and constructing nanocarrier delivery with superior permeability in oral administration application.

## Figures and Tables

**Table 1:** The Zeta potential and hydrodynamic size of the three GNPs. (Error ranges represent standard error of the means).

	Zeta Potential (mV)	Hydrodynamic Size (nm)	Polydispersity
0% MUA GNPs	−4.50 ± 2.25	14.93 ± 1.23	0.231
50% MUA GNPs	−17.52 ± 0.25	14.67 ± 1.50	0.254
100% MUA GNPs	−27.50 ± 0.25	15.14 ± 1.33	0.280

## Supplementary Materials

Supplementary Materials are available online, Figure S1: Cell viability of three GNPs in concentration of 0.01, 0.05 and 0.1 mg/mL, Figure S2. Western Blotting analysis of claudin 5, villin, and ZO-1 protein expression after treated with the three GNPs.
