# Supplementary material for: The High Permeability of Nanocarriers Crossing the Enterocyte Layer by Regulation of the Surface Zonal Pattern
Source: Molecules. 2020 Feb 19;25(4):919. doi: 10.3390/molecules25040919 (PMC7070455; doi:10.3390/molecules25040919)
Supplement: Supplementary file 1 [file molecules-25-00919-s001.zip › molecules-709377-supplementary.docx]

**Supplementary Materials**

**Results:**


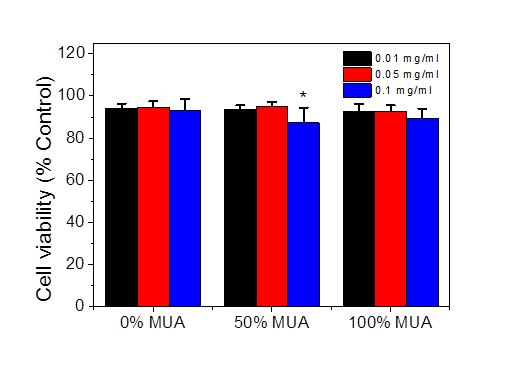


Figure S1. Cell viability of three GNPs in concentration of 0.01, 0.05 and 0.1 mg/mL. * *p<0.05*.


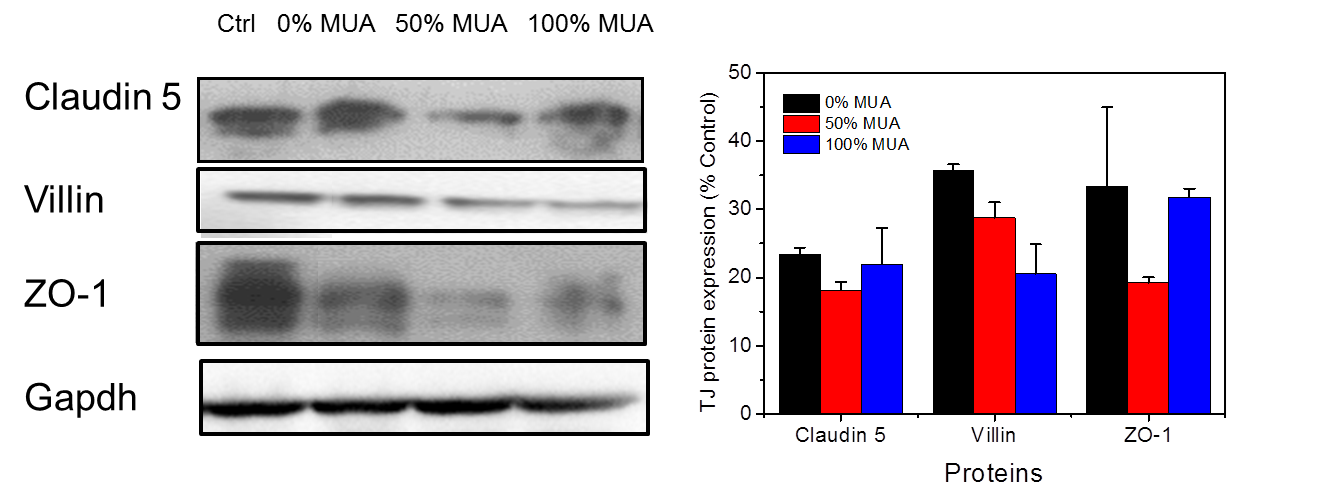


Figure S2. Westernblotting analysis of claudin 5, villin, and ZO-1 protein expression after treated with the three GNPs.
